# Measurement and Study of Electric Field Radiation from a High Voltage Pseudospark Switch

**DOI:** 10.3390/s26020482

**Published:** 2026-01-11

**Authors:** Junou Wang, Lei Chen, Xiao Yu, Jingkun Yang, Fuxing Li, Wanqing Jing

**Affiliations:** 1 Graduate School of China Academy of Engineering Physics, Mianyang 621000, China; wangjunou23@gscaep.ac.cn (J.W.); yangjingkun24@gscaep.ac.cn (J.Y.); 2 Institute of Electronic Engineering, China Academy of Engineering Physics, Mianyang 621000, China; yuxiao@caep.cn (X.Y.); lifuxing@caep.cn (F.L.); jingwanqing@caep.cn (W.J.)

**Keywords:** pulsed discharge, pseudospark switch, near-field scanning, pulsed electric field

## Abstract

The pulsed power switch serves as a critical component in pulsed power systems. The electric radiation generated by switching operations threatens the miniaturization of pulsed power systems, causing significant electromagnetic interference (EMI) to nearby signal circuits. The pseudospark switch’s (PSS) exceptionally fast transient response, a key enabler for sophisticated pulsed power systems, is also a major source of severe EMI. This study investigated the electric field radiation from a high voltage PSS within a capacitor discharge unit (CDU), using a near-field scanning system based on an electro-optic probe. The time-frequency distribution of the radiation was characterized, identifying contributions from three sequential stages: the application of the trigger voltage, the main gap breakdown, and the subsequent oscillating high voltage. During the high-frequency oscillation stage, the distribution of the peak electric field radiation aligns with the predictions of the dipole model, with a maximum value of 43.99 kV/m measured near the PSS. The spectral composition extended to 60 MHz, featuring a primary component at 1.24 MHz and distinct harmonics at 20.14 MHz and 32.33 MHz. Additionally, the impacts of circuit parameters and trigger current on the radiated fields were discussed. These results provided essential guidance for the electromagnetic compatibility (EMC) design of highly-integrated pulsed power systems, facilitating more reliable PSS applications.

## 1. Introduction

**Figure 1:**
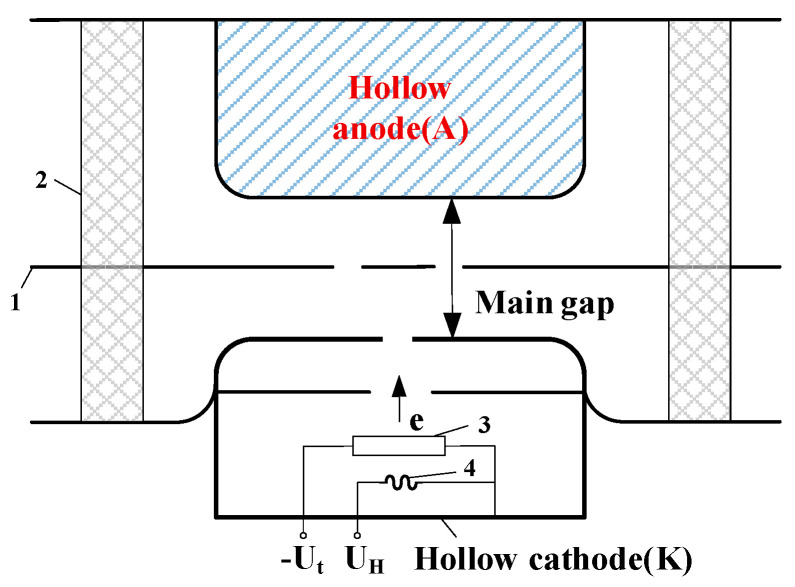
Structure of pseudospark switch (PSS): 1—grid electrode; 2—insulated ceramic; 3—triggering electrode; 4—hydrogen storage.

The performance of modern pulsed power systems in medical, industrial, and military fields hinges on the reliability of key switching devices. Solid-state, gas spark, and vacuum switches are central to this, as their control precision and discharge characteristics are critical for ensuring the dependable operation of compact systems [1,2,3]. The PSS offers a compelling combination of a high voltage and current rise rate (reaching 1600 kV/µs for d*u*/d*t* and 267 kA/µs for d*i*/d*t* [4]), high switching current, and low jitter. This combination of properties makes it an ideal candidate for devices like high-voltage pulse generators, millimeter-wave sources, and plasma ignition systems [1,4,5]. The underlying structure, depicted in Figure 1, features a hollow cathode that facilitates a low-pressure glow discharge. This mechanism is instrumental in confining the discharge plasma, a trait directly attributed to the remarkable current-carrying capacity of the pseudospark switch (PSS) [6,7,8].

In recent years, PSS have advanced toward higher current handling, faster switching speeds, and miniaturization [9,10,11,12,13], driving their implementation in compact circuit designs that are often integrated with control and signal circuitry. For example, reference [9] designed a high-power PSS capable of withstanding voltage up to 40 kV and current up to 25 kA. Yang M et al. have designed several PSS for applied voltage of 2 kV and 10 kV. In this context, PSS are frequently employed in the design of capacitor discharge unit (CDU) discharge circuits. The CDU is a fundamental energy conversion module. By storing electrical energy in a capacitor bank and discharging it via the high-speed switching of the PSS, this unit generates the required high-intensity pulsed currents. The current change rate during PSS discharge has become larger, and with increasing overall device integration, the electromagnetic interference (EMI) problem between high-voltage PSS and signal circuit has become more severe [14,15]. In highly integrated pulsed power devices, the electric field radiation generated during the discharge of a PSS can interfere with the normal operation of low-voltage electronic circuits. The analysis of external electric field radiation characteristics in pulsed power switches is critically important. The development of a distortion-free, contactless, and non-invasive testing method is crucial for accurately obtaining the external electromagnetic spatial distribution generated by PSS pulses.

In previous research and applications, differential probes have been widely used for transient electric field measurement in pulsed power systems due to their excellent common-mode noise rejection and broad frequency response [16,17,18]. However, the physical size of such probes (5–10 cm) limits their deployment in millimeter-scale near-field measurement. Furthermore, studies have found that voltage derating characteristics at high frequencies may affect the accuracy of capturing transient peaks [19], especially in densely wired switch systems where electromagnetic coupling interference further degrades measurement accuracy.

Within the millimeter scale measurement range, the electric field has been found to vary significantly near the switch [20]. Integrated sensors based on the electro-optic effect (Pockels effect) demonstrate unique advantages in compact space measurement. Miniaturized electro-optic sensors using lithium niobate waveguides (125 μm in diameter) can achieve nanosecond-level time resolution and cover field strength ranges from mV/m to kV/m [21]. Their all-dielectric structure avoids electric field disturbance caused by metal components and supports non-intrusive installation [22,23]. Research has indicated that electro-optic probes exhibit a flat response characteristic within the 1 kHz–40 GHz frequency range, enabling the capture of both low-frequency pulse envelopes and fast rise times on the order of 500 ps [24]. In recent years, electro-optic probes have been widely applied in non-intrusive electric field sensing scenarios such as transmission line monitoring [25] and electromagnetic pulse detection [26]. Table 1 compares the performance of three probe types. The electro-optic probe not only exhibits higher sensitivity but also features a much smaller aperture and superior spatial resolution. This enables precise, minimally invasive measurement in the confined near-field region of switches, making it highly suitable for mapping millimeter-scale electric field distributions. However, research specifically targeting the near-field electric field radiation measurement of pulsed power switches remains relatively limited. The near-field radiation distribution and intensity at the millimeter scale have lacked detailed characterization.

We present an investigation into the near-field electric field radiation of a PSS using a custom-built electro-optic measurement system. This work aims to map the radiation intensity, trace its evolution, and identify key influencing factors during switch operation. The paper is organized as follows: the measurement system is described in Section 2; the radiation measurement results and their analysis are reported in Section 3; and discussion on influencing factors is provided in Section 4. Conclusions are summarized in Section 5.

## 2. Electric Field Radiation Measurement Method

### 2.1. Electric Field Radiation Measurement System

**Figure 2:**
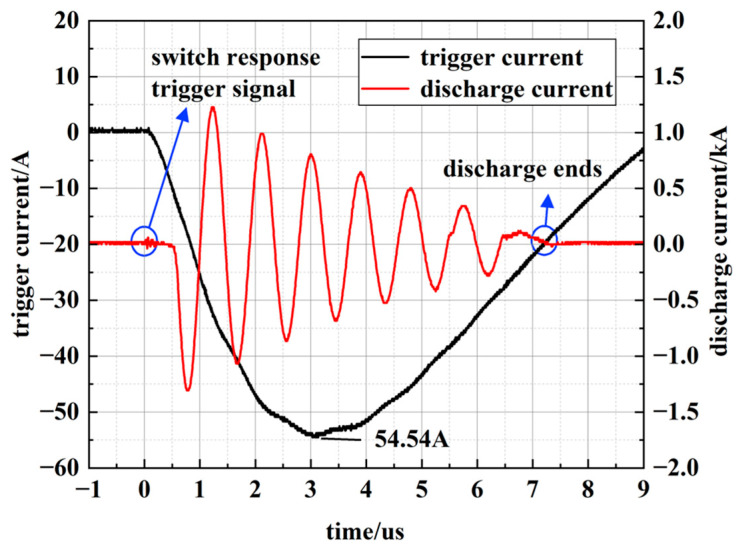
Trigger current waveform.

The PSS used in this experiment required a trigger circuit output with a fast rise time, a peak current exceeding 40 A, and a pulse duration longer than 8 μs. The trigger current waveform is shown in Figure 2.

The measurement principle relies on electro-optic modulation within a lithium niobate (LiNbO_3_) crystal [27,28,29,30]. The system employs a laser source that emits linearly polarized light, which is transmitted through a polarization-maintaining fiber to the sensor head. An external electric field modulates the phase of this light within the optical waveguide. This phase-modulated light is then converted into an intensity-modulated signal through interferometry. Finally, the signal is returned via a single-mode fiber to a photodetector, which produces an electrical output with an amplitude directly proportional to the applied electric field strength, according to the following relation.

(1) $$U_{\mathrm{out}}=A[1+b\cos(\varphi_{0}+\varphi (E))]$$

In the equation, U_out_ is the output electrical signal (mV); *A* represents the optical power loss and the receiver’s photoelectric conversion coefficient; *b* is the extinction ratio of the sensor; *φ*_0_ is the static bias point, determined by the waveguide structure; *φ(**E**)* is the phase change induced by the external electric field ***E*** (kV/m), where *φ(**E**)* = $\frac{E}{E_{\pi }}\pi$, and ***E*_π_** is the half-wave electric field of the sensor.

**Figure 3:**
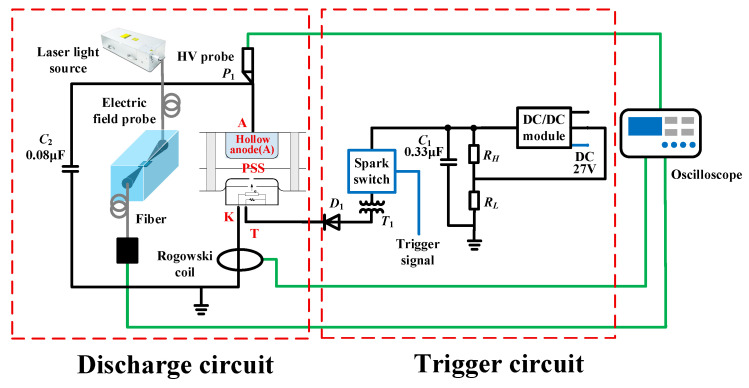
Measurement system for PSS electric field radiation, voltage, and current.

Figure 3 illustrates the experimental measurement system, which consists of a discharge circuit and a trigger circuit. The triggering of the circuit causes the energy storage capacitor *C*_2_ to boost its voltage; following the activation of the discharge circuit, *C*_2_ and the PSS together constitute the CDU. The system is designed to measure the PSS anode voltage, discharge current, and near-field electric field radiation.

In this experiment, a Tektronix P6015A high-voltage probe (*P*_1_) was used for voltage measurements, while a custom-built, calibrated Rogowski coil served as the current probe to meet wide bandwidth requirements. To prevent the triggering system from interfering with electric field measurements, it was placed 50 cm away from the PSS and housed in a galvanized steel shielded enclosure. Under no-load conditions, no electric field was detected at the PSS location using the optical probe when the trigger system was activated.

For grounding, separate connections were established for the high-voltage power supply, the PSS, and the trigger system to maximize current measurement accuracy. Both the trigger and discharge circuits were located inside an electromagnetically shielded chamber. The oscilloscope for data acquisition was placed outside the chamber and connected via optical fiber to eliminate interference from electric field radiation.

**Figure 4:**
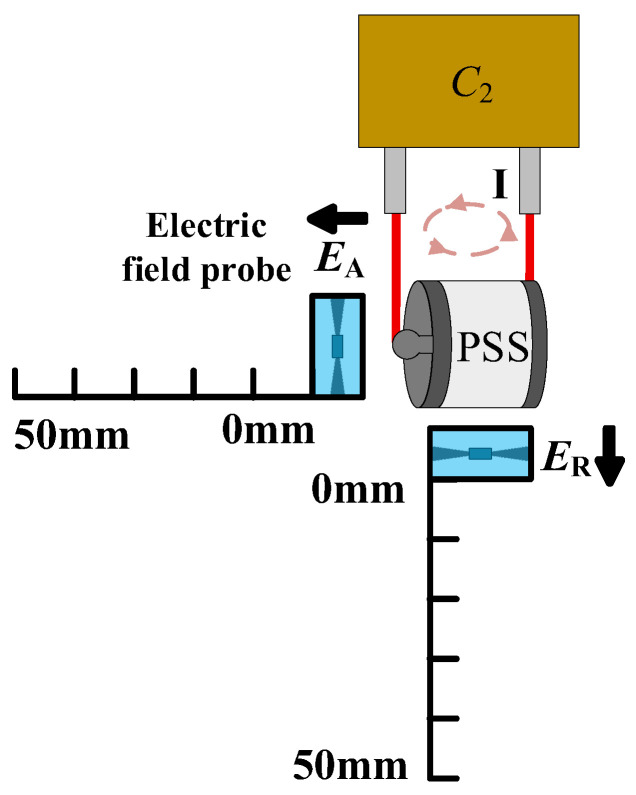
Schematic diagram of the electric field measurement.

As shown in Figure 4, the electric field probe was translated radially from the switch across a range of 0 to 50 mm, using 2 mm increments for the first 20 mm and 5 mm increments thereafter. The probe sequentially measured the axial (***E***_A_) and radial (***E***_R_) field components. The dimensions of the PSS (5 cm height, 3 cm base diameter) and the electric field probe (2.5 cm length, 2 cm × 2 cm cross-section) are provided for scale.

The measurement system is inherently immune to interference due to three key features: its non-contact nature, a well-shielded probe, and optical signal transmission. These attributes enable electric field measurements in close proximity to the PSS surface.

### 2.2. Electric Field Radiation at Different Discharge of PSS

**Figure 5:**
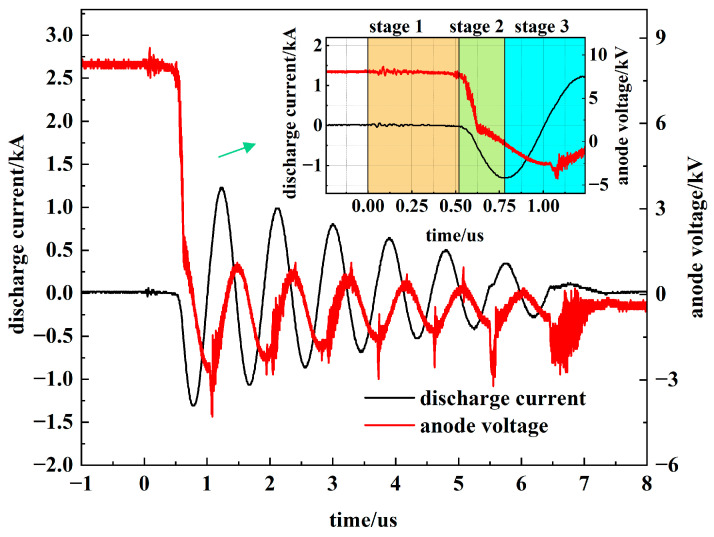
Current and Voltage waveform of PSS discharge. Stage 1—Pre-discharge. Stage 2—Hollow Cathode Discharge. Stage 3—Superdense Glow Discharge.

A PSS features a rapid and complex discharge process characterized by distinct stages. Based on differing discharge characteristics and underlying physical mechanisms, this process is divided into three stages: pre-discharge, hollow cathode discharge, and superdense glow discharge [1]. Figure 5 illustrates the discharge waveforms of the PSS.

Stage 1: Pre-discharge. Positive ions striking the cathode initiate electron emission. During this stage, the anode voltage remains high while the circuit current is negligible. This trigger-induced conduction generates a strong, short-duration, broadband electric field pulse. Specifically, conduction between the trigger electrode and the cathode produces an oscillatory electric field (***E***_1_) with a broad frequency spectrum.

Stage 2: Hollow Cathode Discharge. The accumulation of positive ions in the cathode hole initiates main gap conduction, causing the anode voltage to collapse rapidly as the current rises to its peak. This transition excites a transient electric field with a narrow spectral bandwidth, resulting in short-duration, high-frequency radiation recorded as ***E***_2_.

Stage 3: Superdense Glow Discharge. The main switch gap fully conducts, with both voltage and current exhibiting damped oscillations. The resulting electric field (***E***_3_) synchronously follows the decaying voltage waveform.

**Figure 6:**
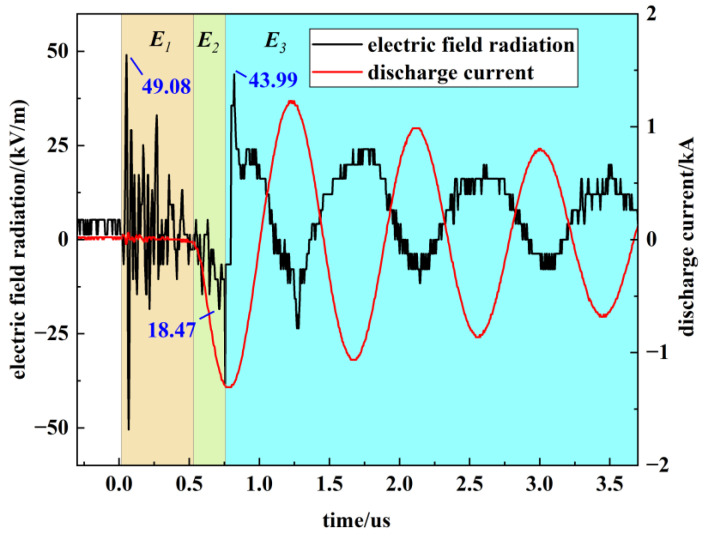
Typical measurement waveform of electric field radiation.

Figure 6 depicts the characteristic electric field radiation, which consists of three distinct segments (***E***_1_, ***E***_2_, ***E***_3_), each corresponding to a specific conduction phase of the PSS.

## 3. Electric Field Measurement Results and Analysis

### 3.1. Electric Field Variation with Distance

The measurement system described in Section 2.1 was employed to characterize the near-field electric radiation of the PSS. Since ***E***_1_ is strongly correlated with the trigger circuit, whereas the main gap discharge more accurately reflects the switch’s external EMI, the analysis focuses on ***E***_2_ and ***E***_3_.

**Figure 7:**
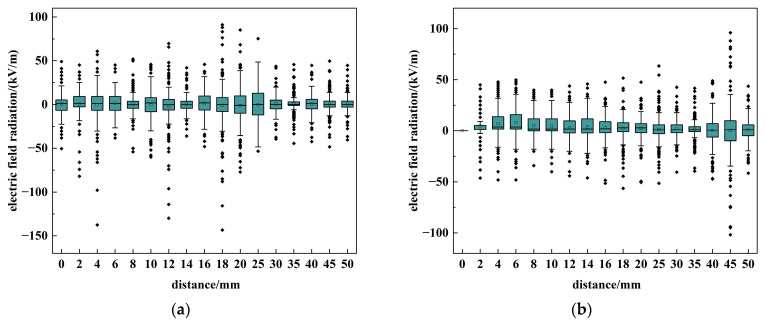
The statistical dispersion of ***E***_2_: (**a**) Axial electric field; (**b**) Radial electric field.

The distribution characteristics of ***E***_2_ are first investigated. Figure 7 presents a box plot of the peak radiation values for ***E***_2_ measured at various distances. The box plot displays the interquartile range (25–75%), with the white square indicating the mean and black diamonds representing outliers.

The experimental results indicate that ***E***_2_ exhibits a symmetric distribution about zero, with nearly identical mean and median values. Furthermore, its amplitude shows negligible attenuation as distance increases, accompanied by a slight divergent trend. This distribution suggests that the ***E***_2_ field constitutes a complex superposition of inductive and radiative components within the near-field zone, which cannot be adequately modeled by simplified equivalents such as a single electric dipole.

**Figure 8:**
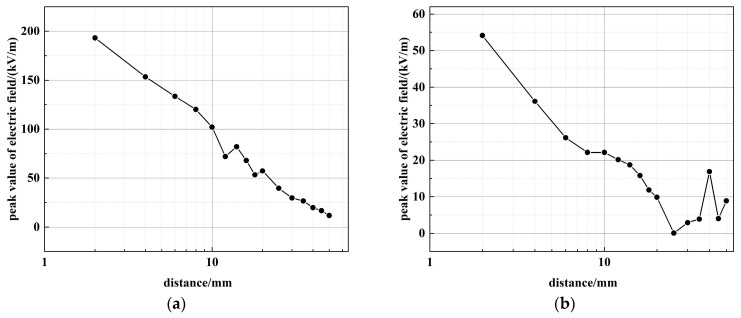
PSS ***E***_3_ decays with distance: (**a**) Axial electric field; (**b**) Radial electric field.

The following discussion addresses the peak values of ***E***_3_. Figure 8 shows the relationship between distance and the peak value of ***E***_3_. The measured peak amplitude of ***E***_3_ demonstrated a clear (1/*x*) decay trend with increasing distance. This observed dependence is consistent with the electric field radiation pattern predicted by the dipole model for electrode structures [31,32].

According to Reference [33], the spark gap resistance formula provided by the Rompe–Weizel model is as follows.

(2) $$r_{s}=\frac{K_{RW}\cdot \delta }{\sqrt{\int_{-\infty }^{t}i{\left(t\prime \right)}^{2}dt\prime }}$$

*i*(*t*) represents the spark switch current. *q* represents the amount of charge carried by the spherical electrode. *δ* represents the length of the dipole. *K_RW_* is a constant depending on discharge atmosphere, pressure, and spark temperature; in case of the atmospheric air, *K_RW_* ≈ 67.4 [V·s^0.5^·m^−1^]. In so doing, *i*(*t*) can be found analytically as shown below [33].

(3) $$i(t)=\frac{q}{\tau }\times \frac{\sqrt{3}}{2}\exp(3\sqrt{3}\cdot \frac{t}{\tau })\cdot {\left[1+\exp(3\sqrt{3}\cdot \frac{t}{\tau })\right]}^{-1.5}$$

Here *τ* called “nominal duration” is effective duration time of spark discharge determined as shown below.

(4) $$\tau =\frac{6\sqrt{3}}{{\left(\frac{V_{c}}{K_{RW}\delta }\right)}^{2}}$$

With the expression of the current known, the electric field intensity can be derived using Maxwell’s equations. Transient electric field *E*(*t*) on a distance *x* from spark point is expressed as follows, *c* denoting speed of light [33].

(5) $$E(t)=\frac{1}{4\pi \varepsilon_{0}q}\sum_{n=0}^{\infty }q_{n}\delta_{n}\cdot [\frac{1}{x^{2}}\cdot i(\frac{t}{\tau }-\frac{x}{c\tau })+\frac{1}{x}\cdot \frac{1}{c\tau }\cdot \frac{\partial i(\frac{t}{\tau }-\frac{x}{c\tau })}{\partial (\frac{t}{\tau }-\frac{x}{c\tau })}]$$

In Equation (5), the 1/*x* term on the right side is dominant at *x* >> *cτ*, which aligns with the experimentally measured results.

**Figure 9:**
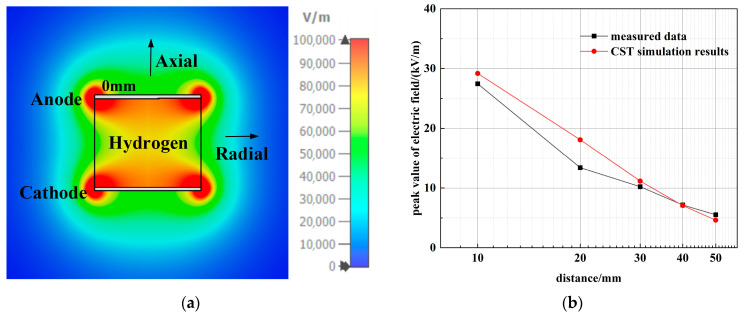
PSS finite-difference time-domain (FDTD) time-domain simulation results: (**a**) PSS ***E***_3_ Axial Peak Electric Field Radiation Distribution; (**b**) Comparison of CST simulation results and measured data.

Correspondingly, the PSS was modeled in CST Studio Suite as an ideal cylinder uniformly filled with hydrogen, utilizing the finite-difference time-domain (FDTD) method. The distribution of the peak ***E***_3_ values on the axial cross-section is shown in Figure 9a, displaying a symmetric radiation pattern that decays outward from the cylinder axis.

A comparison between the simulated and measured radial electric fields is presented in Figure 9b. The simulation reproduces the characteristic 1/*x* decay with distance, in agreement with the measurements, with the residual deviation remaining within acceptable error margins.

### 3.2. Electric Field Variation with Operation Voltage

Applying an overvoltage to a point-plane gap has been shown to reduce pulse rise time [34]. Furthermore, Yambe has established that the relationship between plasma formation time and applied voltage is non-linear [35]. To examine the voltage dependence of ***E***_2_, we measured its duration (from zero to peak current) at a radial distance of 20 mm while increasing the voltage from 5 kV to 9 kV. The resulting ***E***_2_ durations are presented in Table 2.

The experimental results demonstrate a clear positive correlation between the applied voltage and the electric field amplitude. Concurrently, the duration of the ***E***_2_ component exhibits a more complex inverse relationship with the voltage. Specifically, the duration decreases at lower voltages but enters a saturation regime above approximately 7 kV, where it plateaus between 600 ns and 700 ns.

We attribute this to the fact that 7–9 kV represents the optimal operating voltage range for the PSS used in this experiment, within which it maintains stable performance. Although increasing the operating voltage further might reduce the duration of ***E***_2_, it risks damaging both the PSS and the energy-storage capacitor. A similar saturation effect has been reported in the literature: Ref. [36] observes that once the voltage exceeds a certain threshold, the PSS turn-on delay scarcely varies with further voltage increases. Similarly, Ref. [37] shows that in double-pulse triggering studies, fine-tuning trigger parameters yields diminishing returns in improving repetition frequency or shortening delays once the main trigger voltage is raised to a high level. Ding et al. relate this behavior to the hollow-cathode discharge mechanism of the PSS [36]. Pseudospark discharge relies on the penetration of the anode electric field through the cathode hole to form a virtual anode. When the voltage is sufficiently high, the field-penetration process becomes nearly instantaneous; beyond this point, increasing the voltage cannot appreciably shorten this geometrically determined penetration time.

### 3.3. Evolution of the Electric Field Spectrum

**Figure 10:**
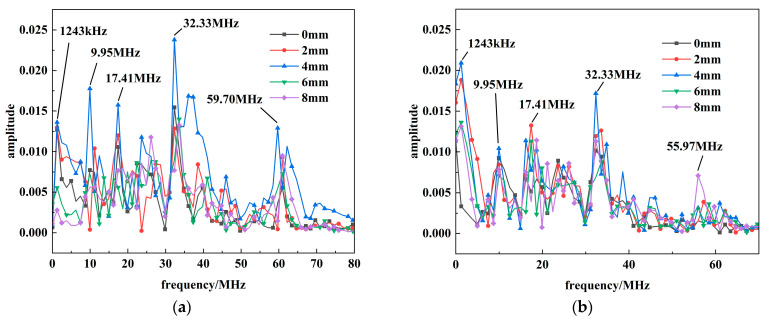
The radiation spectrum of ***E***_2_: (**a**) Axial electric field; (**b**) Radial electric field.

This section characterizes the spectral composition of the ***E***_2_ radiation within 0–8 mm range. The corresponding axial and radial radiation spectra are illustrated in Figure 10.

**Figure 11:**
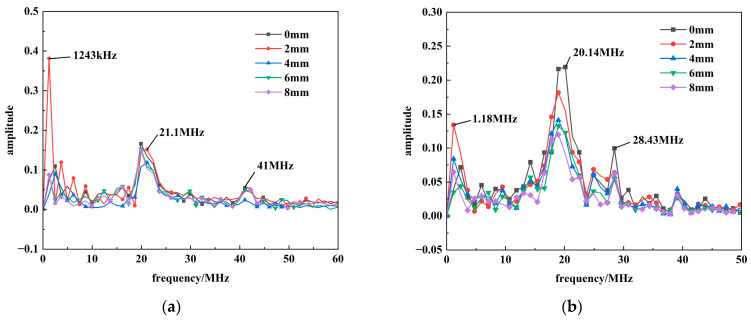
The radiation spectrum of ***E***_3_: (**a**) Axial electric field; (**b**) Radial electric field.

The frequency spectrum of ***E***_2_ is confined to the 0–100 MHz range and initially consists of multiple high-frequency components. Applying a peak threshold of 60% of the maximum amplitude to identify dominant frequencies reveals a clear evolutionary trend as distance increases: lower-frequency components at 1 MHz and 9 MHz progressively diminish, leaving the 30 MHz and 60 MHz bands as the primary constituents. When the distance reaches 30 cm, the 59.97 MHz component attenuates and disappears, leaving only the 32.33 MHz band as the distinctly observable dominant frequency. A similar Fourier transform analysis was performed on ***E***_3_, the results of which are shown in Figure 11.

Beyond the prominent DC component and the sub-2MHz frequencies—which correspond to the voltage/current oscillation—the ***E*_3_** spectrum is characterized by two dominant bands near 20.14 MHz and 28.43 MHz. The DC component itself attenuates with distance. As distance increases, the higher-frequency component around 41 MHz is the first to attenuate to zero, followed subsequently by the attenuation of the 21.1 MHz band.

## 4. Discussion

Pulsed discharge circuit parameters vary considerably depending on the application, leading to distinct operational regimes. Both underdamped and overdamped conditions can arise in practice, each resulting in fundamentally different discharge characteristics and electromagnetic emissions. Notably, altering the CDU circuit parameters, changing the current oscillation state, or adjusting the trigger current affects the rates of current and voltage change (d*i*/d*t* and d*u*/d*t*). These changes consequently influence the breakdown speed of the switch and the intensity of the resulting electric field radiation. This paper further investigates these impacts.

### 4.1. Influence of Circuit Parameters

In specific discharge circuits, an underdamped condition generates a high-frequency oscillatory current. The extreme d*i*/d*t* of this current induces intense overvoltage breakdown, leading to unstable plasma formation and significant reverse current. This phenomenon not only accelerates electrode erosion but also excites complex electromagnetic radiation with rich spectral content and extended duration. Conversely, under an overdamped condition, the current forms a non-oscillatory, single-polarity pulse. The resulting electromagnetic radiation is consequently simpler and more controllable in both the time and frequency domains. These two states are fundamentally distinct in their breakdown speed, plasma dynamics, and ultimate radiation characteristics.

**Figure 12:**
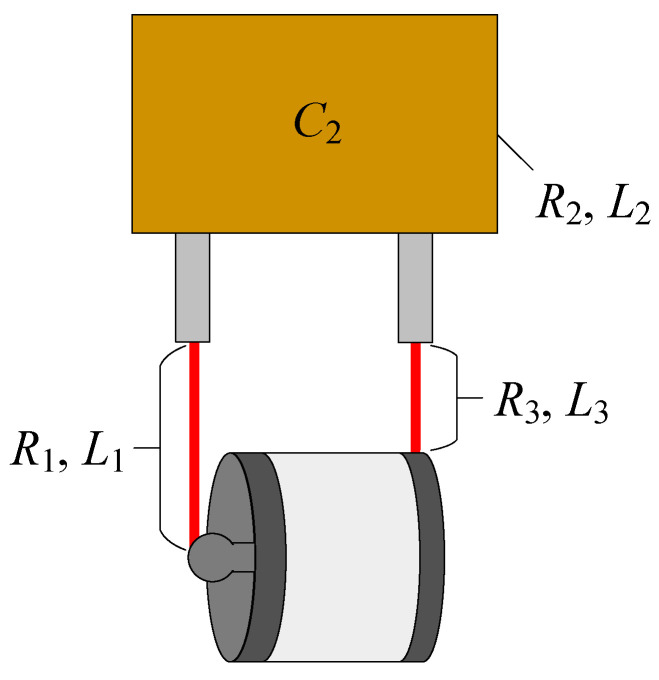
The composition of the parameters *R* and *L*.

The total resistance *R* and inductance *L* of the circuit consist of three distinct elements: the resistance and inductance of the wire connecting the PSS anode to the capacitor (*R*_1_, *L*_1_); the inherent resistance and inductance of the capacitor itself (*R*_2_, *L*_2_), as shown in Figure 12 and the resistance and inductance of the wire linking the PSS cathode to the capacitor (*R*_3_, *L*_3_). A precision impedance analyzer was employed to measure the value of each individual component. Variations in the total circuit parameters *R* and *L* were primarily introduced by adjusting the length of the wire between the PSS anode and the capacitor, thereby modifying its specific contributions *R*_1_ and *L*_1_.

**Figure 13:**
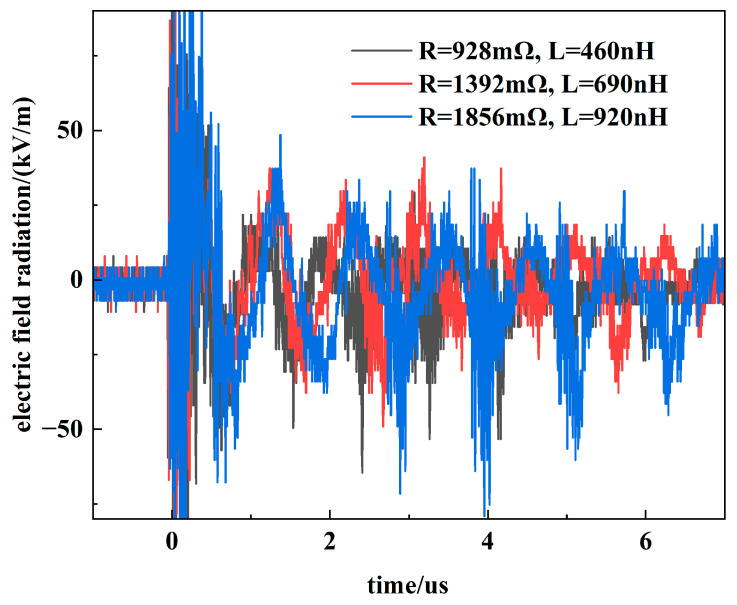
Electric field radiation under different circuit parameters.

The circuit parameters were adjusted from their initial values (*R* = 928 mΩ, *L* = 460 nH) to alter the operational state, while corresponding variations in the electric field radiation were monitored. The measurement results are presented in Figure 13.

Following the adjustment of circuit parameters, a significant increase in the duration of ***E***_2_ was observed, while the peak amplitude of ***E***_3_ remained largely unchanged. Further investigation suggests that this behavior is related to the discharge mechanism of the PSS. An external resistor limits the current rise rate (d*i*/d*t*) in the circuit. Since transitions between discharge phases—particularly from hollow-cathode discharge to superdense glow discharge—typically require the current or space charge to reach a specific threshold, a higher external resistance slows current growth. This, in turn, prolongs the low-current phase before the switch transitions to the next stage [38].

**Figure 14:**
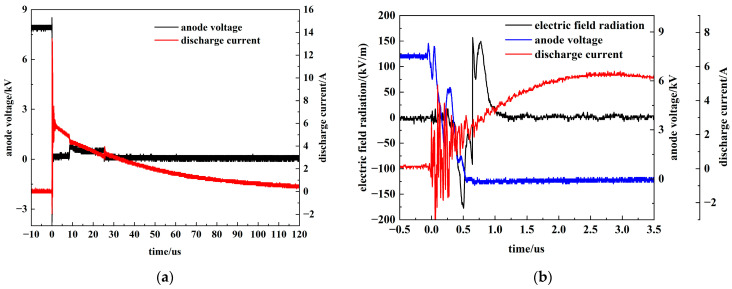
Electric field radiation under overdamped conditions. (**a**) Overdamped voltage and current waveforms; (**b**) The electric field at the instant of PSS discharge under overdamped conditions.

To validate the contrast between these operational states, the circuit was adjusted to an overdamped condition by connecting a 100 Ω resistor in series. Figure 14 displays the electric field radiation measured under this configuration, where Figure 14a illustrates the decaying trend and Figure 14b captures the corresponding voltage, current, and electric field transients at the switching instant.

Under the overdamped condition, the amplitudes of ***E***_2_ and ***E***_3_ decreased markedly, while the ***E***_2_ duration increased. This correlation suggests that the higher circuit impedance restricted the energy available for the initial discharge formation and the subsequent stage 3 oscillations, thereby suppressing the intensity of the radiated electric fields.

### 4.2. Influence of Trigger Current

**Figure 15:**
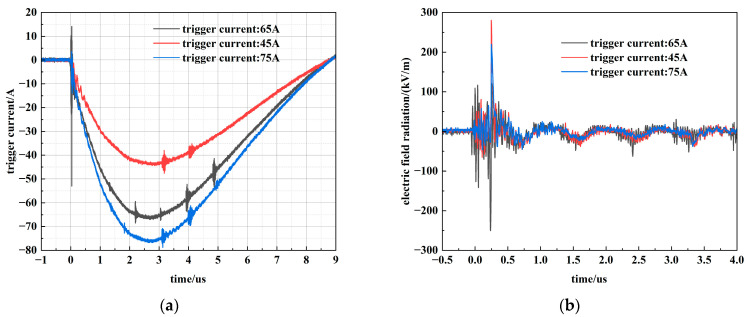
Electric field radiation at different trigger Current. (**a**) Different trigger currents; (**b**) The electric field under different trigger currents.

To further investigate the factors influencing electromagnetic radiation, the trigger circuit was adjusted to vary the PSS trigger voltage, and the resulting changes in electric field radiation were monitored. The optimal trigger current for the PSS is 65 A. To evaluate performance under varied conditions while ensuring normal operation, three trigger current levels—45 A, 65 A, and 75 A—were selected for testing. The corresponding measurement results are presented in Figure 15.

The invariance of the electric field waveforms across the three stages indicates that the resultant radiation intensity is governed primarily by the main discharge circuit dynamics. This finding confirms that the trigger current’s role is predominantly to initiate conduction, with negligible influence on the subsequent radiation, provided the minimum requirements for PSS initiation are met.

### 4.3. Mitigation of Electric Field Radiation

**Figure 16:**
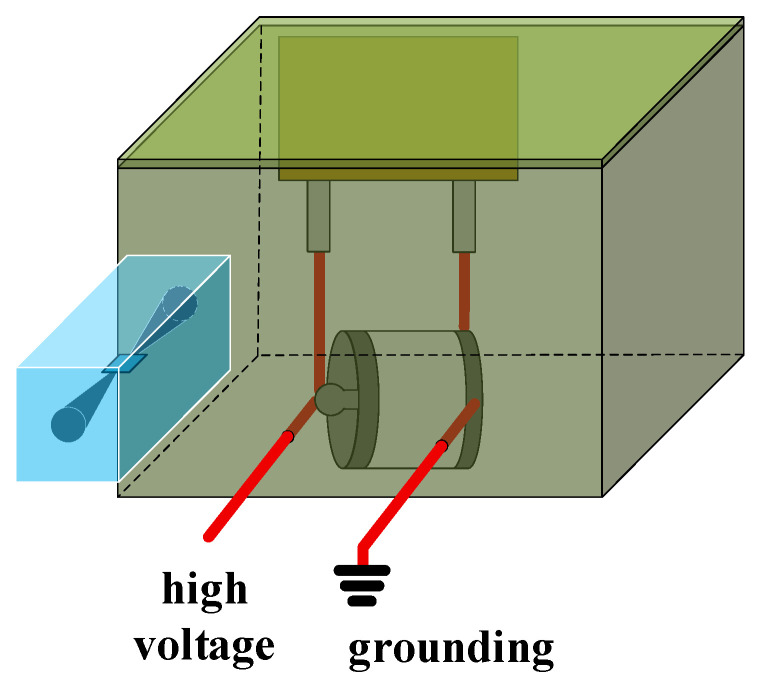
Galvanized steel shielding shell is used to suppress electromagnetic interference (EMI).

Following the discussion on how circuit parameters and trigger current influence the electric field radiation of the PSS, this section briefly introduces radiation mitigation measures. Galvanized steel is commonly used to shield electromagnetic radiation from pulsed power systems. In this experiment, the PSS was placed inside a galvanized steel shielded enclosure, with the anode and cathode leads fed through appropriately sized apertures, as shown in Figure 16. The enclosure cover was secured with screws to ensure maximum shielding integrity.

An optical probe was positioned 10 cm outside the enclosure for measurement. The results show that no electric field radiation from the PSS was detected within the 5–9 kV operating range, indicating that the shielding effectively suppresses external radiation.

However, in tightly integrated pulsed power systems—where multiple high-voltage PSS units and low-voltage components are compactly arranged—space is often insufficient for such enclosures. Therefore, further research is required to address EMI suppression in densely integrated pulsed power systems.

## 5. Conclusions

This study establishes a near-field time-domain electric field measurement system based on an electro-optic probe. The system was used to characterize the axial and radial electric field radiation from a high-voltage PSS, followed by a comprehensive time-frequency analysis. The research focuses on the radiation characteristics across three key stages: trigger-induced conduction, main gap breakdown, and the subsequent oscillating pulsed voltage phase. The effects of distance, voltage, and circuit parameters were systematically investigated. The results demonstrate the following:

1. PSS discharge radiation comprises three distinct components: a highly oscillatory field generated during trigger-cathode conduction; a short-duration, high-frequency field resulting from main gap breakdown; and an exponentially decaying sinusoidal field driven by the underdamped pulsed discharge current.
2. In Stage 2, the peak amplitude of ***E***_2_ increases with the applied voltage following an exponential growth trend. Concurrently, the duration of ***E***_2_ follows an exponential decay pattern, eventually stabilizing between 600 ns and 700 ns as the voltage reaches the 7–9 kV range.
3. In Stage 3, the peak amplitude of ***E***_3_ is inversely proportional to the measurement distance, which is consistent with the predictions of the electric dipole radiation model and has been validated through simulations. Axial radiation contains high-frequency components at 21.1 MHz and 41 MHz, while radial radiation exhibits distinct components at 20.14 MHz and 28.43 MHz.
4. Increasing the resistance and inductance of the discharge circuit prolongs the duration of ***E***_2_ and attenuates the amplitude of ***E***_3_. In contrast, variations in the trigger current have no measurable effect on the electric field radiated by the PSS. Furthermore, a shielded enclosure made of galvanized steel effectively suppresses the PSS electric field radiation.

## Figures and Tables

**Table 1:** Performance comparison of various probe.

Probe Type	Aperture Size	Spatial Resolution	Sensitivity (1 GHz)
Differential probes	5 mm	8 mm	−45 dBm
Miniature ring probe	1 mm	1.5 mm	−65 dBm
Electro-optic probe	50 μm	100 μm	−30 dBm

**Table 2:** The peak value and duration of ***E***_2_.

Applied Voltage (kV)	Peak Value (kV/m)	Duration (ns)
5	17.131	2400
5.5	24.076	2000
6	22.687	1200
6.5	30.095	800
7	42.133	700
7.4	45.157	628
7.8	48.895	630
8.2	44.224	656
8.5	49.541	600
9	49.863	600

## Data Availability

The original contributions presented in this study are included in the article. Further inquiries can be directed to the corresponding author.

## Abbreviations

The following abbreviations are used in this manuscript:

EMI	Electromagnetic Interference
PSS	Pseudospark Switch
CDU	Capacitor Discharge Unit
EMC	Electromagnetic Compatibility
